# Mechanical Implications of Estrogen Supplementation in Early Postmenopausal Women

**DOI:** 10.1002/jbmr.33

**Published:** 2010-01-29

**Authors:** Felix W Wehrli, Chamith S Rajapakse, Jeremy F Magland, Peter J Snyder

**Affiliations:** 1 Department of Radiology, Laboratory for Structural NMR Imaging, University of PennsylvaniaPhiladelphia, PA, USA; 2 Department of Medicine, Division of Endocrinology, Diabetes & Metabolism, University of PennsylvaniaPhiladelphia, PA, USA

**Keywords:** MRI, trabecular bone, cortical bone, structure, finite element, menopause, estrogen

## Abstract

Whereas the structural implications of drug intervention are well established, there are few data on the possible mechanical consequences of treatment. In this work we examined the changes in elastic and shear moduli (EM and SM) in a region of trabecular bone in the distal radius and distal tibia of early postmenopausal women on the basis of MRI-based micro-finite-element (µFE) analysis. Whole-section axial stiffness (AS) encompassing both trabecular and cortical compartments was evaluated as well. The study was conducted on previously acquired high-resolution images at the two anatomic sites. Images were processed to yield a 3D voxel array of bone-volume fraction (BVF), which was converted to a µFE model of hexahedral elements in which tissue modulus was set proportional to voxel BVF. The study comprised 65 early postmenopausal women (age range 45 to 55 years), of whom 32 had chosen estrogen supplementation (estradiol group); the remainder had not (control group). Subjects had been scanned at baseline and 12 and 24 months thereafter. At the distal tibia, EM and SM were reduced by 2.9% to 5.5% in the control group (*p* < .05 to <.005), but there was no change in the estradiol subjects. AS decreased 3.9% (4.0%) in controls (*p* < .005) and increased by 5.8% (6.2%) in estradiol group subjects (*p* < .05) at 12 (24) months. At the distal radius, EM and SM changes from baseline were not significant, but at both time points AS was increased in estradiol group subjects and decreased in controls (*p* < .005 to <.05), albeit by a smaller margin than at the tibia. EM and SM were strongly correlated with BV/TV (*r*^2^ = 0.44 to 0.92) as well as with topologic parameters expressing the ratio of plates to rods (*r*^2^ = 0.45 to 0.82), jointly explaining up to 96% of the variation in the mechanical parameters. Finally, baseline AS was strongly correlated between the two anatomic sites (*r*^2^ = 0.58), suggesting that intersubject variations in the bone's mechanical competence follows similar mechanisms. In conclusion, the results demonstrate that micro-MRI-based µFE models are suited for the study of the mechanical implications of antiresorptive treatment. The data further highlight the anabolic effect of short-term estrogen supplementation. © 2010 American Society for Bone and Mineral Research.

## Introduction

The notion that besides bone mineral density, parameters related to architecture at the macro- and microstructural levels,(1) as well as mineral and matrix chemistry,(2) collectively summarized under the term *bone quality*,(3) determine overall bone strength is now generally accepted. There is also consensus that changes in many of the parameters occurring during aging and disease progression (or regression in response to treatment) can affect the bone's mechanical behavior in a manner independent of the measured changes in material density.(4)

While initially confined to measurements in small specimens, advances in imaging technology, both computed tomography (CT) and magnetic resonance imaging (MRI), now allow detailed models to be reconstructed from high-resolution 3D images of both trabecular and cortical microarchitecture acquired in vivo in patients. These and related imaging technologies and their merits and current limitations have been reviewed extensively (see, for example, refs. (5–7)). On the basis of such imaging data, it has become possible to derive a host of structural measures expressing properties of the trabecular network in terms of scale, topology, and orientation, known from specimen studies to be relevant to the bone's mechanical properties.(8,9) Similarly, macro- and microstructural properties can be assessed for cortical bone, such as cortical thickness and cross-sectional area, via direct quantification of these structural measures on the basis of MRI(6) or CT.(10) Microstructural parameters such as porosity have been shown to be obtainable indirectly, for example, via MRI-based quantification of bone water.(11)

There are a number of reports showing associations between structural measures at peripheral sites such as the radius or calcaneus and vertebral or femur fracture status.(12–15) It also has been demonstrated recently that modern imaging technologies, notably micro-magnetic resonance imaging (µMRI), whole-body multislice CT, and high-resolution peripheral quantitative computed tomography (HR-pQCT), are able to detect age-related loss of trabecular network integrity,(16) as well as structural changes in response to drug intervention,(17–20)

Whereas there is evidence that certain structural features make trabecular bone more prone to failure (eg, rodlike versus platelike(21) or increased degree of structural anisotropy(22)), only recently, capabilities have become available to identify such structural properties on the basis of in vivo images and quantify changes serially over time in human subjects.(17,18,20) However, even though empirically established associations are known between biomechanical and structural parameters, the latter largely represent surrogates for strength. Since mechanical testing in vivo is not possible, alternate approaches have been pursued to estimate strength on the basis of image-derived finite-element meshes (see, for example, ref. (23)). Micro-finite element (µFE) models established from high-resolution µCT images yielded mechanical constants in excellent agreement with those obtained by mechanical testing.(24) Further, strong correlations with actual measures of elastic moduli and image-derived values have been shown recently to be obtainable even after downsampling the µCT images to lower resolution and superposition of noise to simulate in vivo imaging conditions.(25) These and other data instill significant confidence that meaningful biomechanical data are obtainable from in vivo image-based FE models.

Early work by Muller and Ruegegger, based on simulated atrophy of images acquired at in vivo resolution by peripheral CT in a cadaver specimen, subjected to µFE computation of elastic moduli, showed that a relatively small amount of bone loss can have disproportionately large mechanical consequences.(26) The work suggested the feasibility of evaluating disease and aging-related effects on the bone's mechanical behavior. Van Rietbergen and colleagues examined the potential of µMRI to quantify the effects of drug intervention in patients treated with a selective estrogen receptor modulator.(27) A recent article showed in 10 hypogonadal subjects that 2-year treatment with testosterone resulted in significant increases in some elastic and shear moduli,(28) computed from a region of trabecular bone in the distal tibial metaphysis, commensurate with changes in topology of the trabecular network reported earlier.(18)

The preceding study is of limited scope in terms of both the number of subjects studied and the size of the imaging volume used for construction of the µFE model. In the present work, we have performed µFE analysis on MR data in the distal tibia and radius from a recent structural imaging study in early postmenopausal women who elected estrogen supplementation compared with those who did not during a 2-year period. The image data were reprocessed yielding grayscale FE models of both central trabecular subregions used for multidirectional estimation of Young's and shear moduli, as well as whole-section simulated compressive testing, including both trabecular and cortical bone compartments, at three time points.

## Materials and Methods

### Subjects and image acquisition

This work is based on imaging data from a recently completed study in the authors' laboratory(20) involving 65 early postmenopausal women, 45 to 55 years of age, who had been recruited for the study. Thirty-two of the women had chosen estrogen supplementation (estradiol group); the remainder had not (control group). µMRI in the metaphysis of the distal radius and distal tibia had been performed at 137 × 137 × 410 µm^3^ voxel size at baseline and 12 and 24 months to determine the temporal changes in the trabecular architecture. The imaging volume was 70 × 40 × 13 mm^3^ (scan time 12 minutes) and 70 × 50 × 13 mm^3^ (scan time 16 minutes) for the distal radius and distal tibia, respectively, with the third dimension representing the axial direction. Full details of the subject characteristics and image acquisition protocol are given in ref. (20).

### Image processing

Images from the prior study were reprocessed using more advanced algorithms. The raw images were corrected for translational displacements during the scan owing to involuntary subject motion with the aid of navigator echo data acquired simultaneously with the image data.(29) Motion-corrected *k*-space data then were Fourier-transformed to yield 32 image slices. Some examinations did not provide images of sufficient quality for analysis because of artifacts caused by subject motion during scanning that could not be removed via navigator correction (eg, rotational motion) or wrap-around artifacts resulting from field-of-view restrictions for some subjects with particularly large wrists and ankles. These images were excluded from analysis by following a procedure described previously.(20,30) Further, if the common image volume between the three time points was less than 2.5 mm in the axial direction (owing to registration errors), those images also were excluded from the analysis. In summary, the number of subjects who provided distal tibia data with adequate image quality for the baseline to 12-month (24) interval was 28 (23) for the control group and 23 (19) for the estradiol group. Similarly, the corresponding numbers for the distal radius were 23 (14) and 13 (12), respectively.

**Fig. 1:**
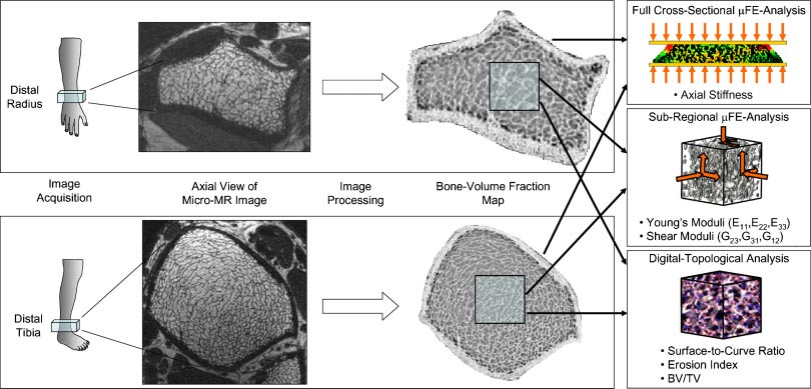
Image acquisition and processing flow to compute full cross-sectional axial stiffness and subregional mechanical and topologic parameters of trabecular bone from µMRI images of distal radius and distal tibia.

The preprocessed images then were subjected to a cascade of processing steps before being used as input to the mechanical and topologic analysis (Fig. 1). First, image intensity variations across the volume produced by inhomogeneous sensitivity of the MR receive coil were corrected using a local thresholding algorithm.(31) The grayscale voxel values of the intensity-corrected images then were scaled linearly to cover the range from 0% to 100%, with pure marrow and pure bone having minimum and maximum values, respectively. We refer to the resulting 3D array as the *bone-volume fraction* (BVF) *map*, with individual voxel values representing the fraction of the voxel occupied by bone (ie, BV/TV). Subsequently, the BVF maps were subjected to two different processing paths in preparation for the full cross-sectional and subregional analysis. To enable full cross-sectional analysis, the soft tissue was first segmented out from bone by delineating the periosteal boundary using an operator-guided region-selection program developed in-house.(32) Finally, common 5-mm sections along the bone's axial direction were extracted from the images at the three time points for each subject. To prepare the images for subregional analysis, BVF maps were first *sinc*-interpolated by a factor of 3 in all three image coordinate axes to improve apparent resolution, yielding 45.7 × 45.7 × 137 µm^3^ voxels. The 12- and 24-month repeat images of each subject then were registered in 3D(33) to match the corresponding baseline volume using trilinear interpolation. Finally, 10 × 10 × 5 mm^3^ cuboid subvolumes were extracted from the center of the trabecular bone (TB) region for analysis.

### Grayscale-based µFE modeling

To create a finite-element mesh, each voxel in the BVF map was converted directly to a hexahedral (brick) finite element with dimensions corresponding to the voxel size,(34) The bone tissue material properties were chosen as isotropic and linearly elastic, with each element's Young's modulus (YM) set to be linearly proportional to the voxel's BV/TV value such that YM = 15 GPa × BVF, while Poisson's ratio was kept constant at 0.3 for all elements. A partial threshold was applied by setting finite elements with BVF < 20% to zero to remove the contributions from image noise.

### Subregional µFE analysis

To estimate the mechanical properties of the cuboid subregion from the generated µFE model, Young's and shear moduli, scalar quantities that express the relationship between stress and strain, were determined by simulating compression and shear tests on the µFE model by imposing different boundary conditions on the eight surfaces of the modeled bone region. The Young's moduli, representing the stiffness along each of the three principal axes (*E*_11_, *E*_22_, and *E*_33_) of the coordinate system (*x*, *y*, and *z*), were found by performing three simulated “compressive” tests along each of the three orthogonal axes.(34,35) Since the patients had been imaged in the supine position with the tibia's long axis parallel to the magnet bore, the scanner coordinate system was essentially collinear with the anatomic coordinate frame. Thus the subscripts 1, 2, and 3 correspond to anteroposterior, mediolateral, and inferosuperior directions, respectively. The value of *E*_11_, for example, was obtained by applying a small simulated displacement (compressive strain of approximately 1%) along the *x* direction to all nodes on one of the boundary surfaces perpendicular to the *x* axis in such a manner that the nodes on the opposite surface were restrained along the *x* direction while lateral displacements remained unrestrained. The unknown displacements at each node then were found by minimizing the total strain energy of the system. Subsequently, the resulting stress along the *x* direction was calculated by summing the *x* components for the reaction force and dividing by the surface area. Finally, *E*_11_ was estimated as the ratio of calculated stress to the applied strain. *E*_22_ and *E*_33_ also were obtained analogously. Using a similar approach, shear moduli (*G*_23_, *G*_31_, and *G*_12_), expressing the ratio of shear stress to shear strain of the modeled region, were estimated by simulating shear displacements applied to nodes on opposite surfaces along antiparallel directions.

### Full cross-sectional µFE analysis

Axial stiffness of the distal radius and distal tibia was estimated via µFE analysis of the full FE mesh comprising both trabecular and cortical bone compartments generated as described earlier. First, simulated compression was applied along the bone's longitudinal axis by applying a constant displacement (∼1% strain) to all FE nodes in the proximal face of the FE mesh while keeping those in the distal face constrained.(36) The µFE system then was solved as described earlier, yielding a 3D strain map. Finally, the axial full cross-sectional modulus was obtained as the ratio of the resulting stress on the proximal face to the applied strain.

### Digital-topologic analysis

In order to evaluate parameters of scale and topology of the trabecular bone network, digital-topologic analysis (DTA)(37) was performed on the same cuboid subvolumes of trabecular bone used for µFE analysis. In brief, the *sinc*-interpolated BVF maps obtained as described earlier were binarized such that bone and background voxels were assigned values of 1 and 0, respectively, using an empirically optimized threshold level of 20% of pure bone intensity.(38) The binary volumes then were subjected to a topology-preserving thinning algorithm(39) yielding the 3D skeleton of the trabecular bone network. Subsequently, topologic classification was performed by identifying each voxel as belonging to a surface, curve, or mutual junctions between these entities.(37) Finally, the composite topologic parameters surface-to-curve ratio (S/C) and erosion index (EI) were computed from the DTA maps of the skeleton.(37) BV/TV was computed as described in ref. (31).

### Statistical analysis

The statistical analysis was analogous to the one outlined in the parent article,(20) based on multivariate analysis of variance (MANOVA). Following determination that the data were normally distributed, MANOVA was performed with time (repeated) and treatment (nonrepeated) factors along with inclusion of the treatment × time cross-product to assess second-order interaction. When a response variable was associated with significant treatment × time interaction, simple effects over time were assessed within each treatment groups using two-sided paired *t* tests. Between-group differences in the changes from baseline were evaluated by unpaired *t* tests. Intersite correlation of full cross-sectional stiffness, as well as correlations between mechanical and topologic parameters of trabecular bone, were evaluated by least-squares regression in terms of the Pearson correlation coefficient. All statistical analyses were performed using JMP Discovery Software, Version 7.02, (SAS Institute, Inc., Cary, NC), with *p* < .05 indicating statistical significance.

## Results

### Subregional µFE analysis

**Fig. 2:**
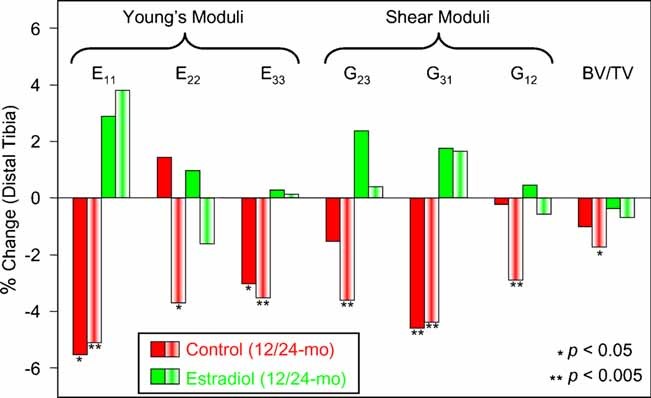
Within-group mean temporal changes (%) in subregional mechanical parameters and BV/TV of distal tibia from baseline to 12 and 24 months. The *p* values indicate the significance of the computed changes. Between-group changes from baseline were not significant.

The mean values of Young's and shear moduli, as well as BV/TV, all computed from matching trabecular bone subvolumes in the distal tibia at all three time points, as well as the relative changes from baseline, are summarized in Tables 1 and 2. The relative temporal changes in parameters are charted in Fig. 2. The subregional mechanical parameters did not show any significant change from baseline to the two follow-up time points in the estradiol group, suggesting that estrogen supplementation preserves the integrity of the trabecular network, as shown in some of our prior work.(20) On the other hand, in the control subjects, three of the mechanical parameters suggest significant reduction already after 12 months (3.0% to 5.5%, *p* = .01 to .002), whereas the change in BV/TV was not significant. At 24 months, all six mechanical parameters were reduced significantly in the control group relative to baseline values (2.9% to 5.1%, *p* = .03 to .002), at which point the reduction in BV/TV also became significant (1.8%, *p* < .05). None of the subregional between-group differences of changes from baseline were significant.

**Table 1:** Young's Moduli (*E*_11_, *E*_22_, *E*_33_) and Shear Moduli (*G*_12_, *G*_13_, *G*_23_), Cross-Sectional Stiffness (All in MPa), and BV/TV (%) at the Distal Tibia Derived from µMRI Images in Control Subjects at Baseline and 12 and 24 Months, Including Relative Changes of Mean Values from Baseline to Both Follow-Up Time Points

	Mean value			Baseline to 12 mos.		Baseline to 24 mos./	
			
Parameter	Baseline	12 mos.	24 mos.	Relative change (%)	*p*	Relative change (%)	*p*
*E*_11_	243 ± 75	228 ± 70	233 ± 51	−5.53	.006	−5.14	.005
*E*_22_	199 ± 38	202 ± 46	192 ± 39	1.44	.309	−3.71	.018
*E*_33_	827 ± 164	799 ± 151	805 ± 126	−3.01	.013	−3.55	.003
*G*_12_	155 ± 30	155 ± 31	151 ± 28	−0.21	.850	−2.93	.005
*G*_13_	181 ± 45	172 ± 42	175 ± 32	−4.60	.002	−4.41	.002
*G*_23_	103 ± 24	102 ± 25	101 ± 21	−1.51	.231	−3.64	.005
Stiffness	1222 ± 144	1184 ± 150	1156 ± 126	−3.89	.0004	−4.03	.0011
BV/TV	11.0 ± 1.2	10.9 ± 1.1	10.9 ± 1.0	−0.99	.071	−1.76	.026

**Table 2:** Subregional Young's Moduli (*E*_11_, *E*_22_, *E*_33_), Shear Moduli (*G*_12_, *G*_13_, *G*_23_), Cross-Sectional Stiffness (All in MPa), and BV/TV (%) at the Distal Tibia, Derived from µMR Images in Estradiol Subjects at Baseline and 12 and 24 Months, Including Relative Changes of Mean Values from Baseline to Both Follow-Up Time Points

	Mean value			Baseline to 12 mos.		Baseline to 24 mos.	
			
Parameter	Baseline	12 mos.	24 mos.	Relative change (%)	*p*	Relative change (%)	*p*
*E*_11_	230 ± 68	231 ± 60	241 ± 59	2.89	.49	3.79	.44
*E*_22_	193 ± 38	194 ± 36	194 ± 30	0.96	.66	−1.63	.60
*E*_33_	840 ± 149	838 ± 140	859 ± 128	0.27	.90	0.10	.97
*G*_12_	153 ± 26	153 ± 24	156 ± 19	0.45	.79	−0.58	.80
*G*_13_	179 ± 45	179 ± 39	184 ± 40	1.75	.55	1.64	.61
*G*_23_	97 ± 20	98 ± 18	99 ± 16	2.38	.32	0.37	.91
Stiffness	1158 ± 139	1218 ± 116	1177 ± 80	5.76	.03	6.22	.03
BV/TV	11.2 ± 1.1	11.1 ± 1.0	11.3 ± 1.0	−0.35	.68	−0.26	.81

For the distal radius (Tables 3 and 4), the temporal changes in mean values of subregional mechanical parameters did not reach statistical significance except for *E*_22_ (at 12 months) and *G*_23_ (at 24 months) for the estradiol group, for which 6.1% ± 2.2% (*p* = .02) and 3.8% ± 1.5% (*p* = .04) increases were observed, respectively. Further, the detected change in BV/TV was nonsignificant for both groups at both time points. Again, at this site, there was no significant difference in the temporal changes between groups.

**Table 3:** Subregional Young's Moduli (*E*_11_, *E*_22_, *E*_33_) and Shear Moduli (*G*_12_, *G*_13_, *G*_23_), Cross-Sectional Stiffness (All in MPa) and BV/TV (%) at the Distal Radius Derived from µMR Images in Control Subjects at Baseline and 12 and 24 Months, Including Relative Changes of Mean Values from Baseline to Both Follow-Up Time Points

	Mean value			Baseline to 12 mos.		Baseline to 24 mos.	
			
Parameter	Baseline	12 mos.	24 mos.	Relative change (%)	*p*	Relative change (%)	*p*
*E*_11_	302 ± 84	290 ± 67	286 ± 62	2.89	.49	3.79	.44
*E*_22_	404 ± 99	391 ± 82	373 ± 73	0.96	.66	−1.63	.60
*E*_33_	838 ± 117	813 ± 102	824 ± 127	0.27	.90	0.10	.97
*G*_12_	249 ± 47	242 ± 38	235 ± 37	0.45	.79	−0.58	.80
*G*_13_	211 ± 40	203 ± 33	201 ± 31	1.75	.55	1.64	.65
*G*_23_	170 ± 49	161 ± 40	152 ± 35	2.38	.32	0.37	.91
Stiffness	1547 ± 206	1542 ± 207	1487 ± 188	−3.57	.002	−3.65	.03
BV/TV	9.8 ± 1.2	9.8 ± 1.0	10.2 ± 1.7	−0.35	.68	−0.26	.81

**Table 4:** Subregional Young's Moduli (*E*_11_, *E*_22_, *E*_33_) and Shear Moduli (*G*_12_, *G*_13_, *G*_23_), Cross-Sectional Stiffness (All in MPa), and BV/TV (%) at the Distal Radius Derived from µMR Images in Estradiol Subjects at Baseline and 12 and 24 months, Including Relative Changes of Mean Values from Baseline to Both Follow-Up Time Points

	Mean value			Baseline to 12 mos.		Baseline to 24 mos.	
			
Parameter	Baseline	12 mos.	24 mos.	Relative change (%)	*p*	Relative change (%)	*p*
*E*_11_	281 ± 82	310 ± 111	327 ± 132	2.89	.490	3.79	.442
*E*_22_	376 ± 94	407 ± 114	409 ± 120	0.96	.655	−1.63	.596
*E*_33_	813 ± 88	799 ± 108	823 ± 133	0.27	.901	0.10	.966
*G*_12_	230 ± 42	239 ± 53	245 ± 60	0.45	.792	−0.58	.795
*G*_13_	197 ± 39	207 ± 53	212 ± 62	1.75	.554	1.64	.647
*G*_23_	157 ± 50	172 ± 65	177 ± 74	2.38	.322	0.37	.907
Stiffness	1382 ± 384	1477 ±163	1486 ± 145	2.52	.024	3.16	.065
BV/TV	9.4 ± 0.9	10.7 ± 3.6	12.7 ± 5.6	−0.35	.677	−0.26	.811

### Full cross-sectional µFE analysis

**Fig. 3:**
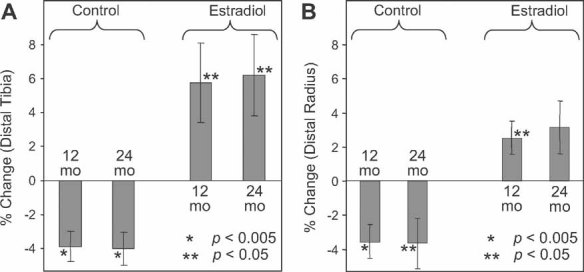
Within-group mean temporal changes (%) in full cross-sectional axial stiffness of (*A*) distal tibia and (*B*) distal radius from baseline to the two follow-up time points. Error bars represent standard error of mean changes. Intergroup differences in the temporal changes were significant at both follow-up time points and for both anatomic sites (*p* < .005). For details, see text.

Tables 1 through 4 also provide mean values and relative temporal changes in full cross-sectional axial stiffness at the two anatomic sites from baseline to the follow-up time points. Figure 3 displays the fractional changes from baseline to 12 and 24 months. Estrogen supplementation increased whole-section axial stiffness after 12 and 24 months of treatment at both anatomic sites. At the tibia, the increases were 5.8% and 6.3% (both *p* = .03) at 12 and 24 months, respectively. At the radius, axial stiffness increased 2.5% (*p* = .02) at 12 months and 3.2% at 24 months, but the latter effect did not quite reach significance (*p* = .065). In control subjects, the data further revealed substantial and highly significant reductions in axial stiffness (3.9% and 4.0%, *p* = .0004 and .001 at 12 and 24 months, respectively) at the tibia and 3.6% and 3.7% (*p* = .002 and .03) at the radius at the two time points.

**Fig. 4:**
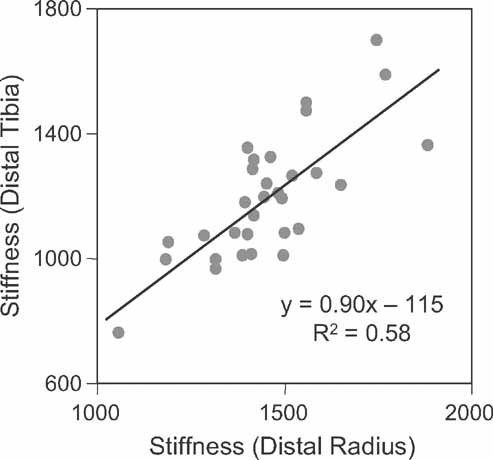
Comparison of full cross-sectional stiffness (MPa) of distal tibia and distal radius computed from µMR-based µFE analysis at baseline.

The intergroup differences in the observed temporal changes in axial stiffness were highly significant at both follow-up time points and for both anatomic sites. However, the values were greater for the distal tibia than for the distal radius at 12 months (9.7% ± 2.5%, *p* = .0001, versus 6.1% ± 1.5%, *p* = .0003). At 24 months, the corresponding intergroup changes were 10.2% ± 2.3% (*p* = 0.0002) at the tibia versus 6.8% ± 2.1% (*p* = 0.004) at the radius. Further, the full cross-sectional axial stiffness values at tibial and radial sites were highly correlated with each other, indicating the systemic nature of these parameters (Fig. 4).

### Relationship between mechanical and topologic parameters

Strong correlations were observed between the mechanical and composite topologic parameters (S/C and EI) computed from the same trabecular bone subvolumes at the distal tibia (Table 5). The strength of the correlations differed for the three directions of Young's moduli, being largest for the axial direction (*E*_33_). Further, the predictive ability of two-parameter linear regression models involving BV/TV and either S/C or EI improved relative to a single predictor. Similar trends were observed for the correlations between mechanical and composite topologic parameters computed from the distal radius. However, the correlations (*r*^*2*^ ranging from 0.24 to 0.89) generally were somewhat weaker than those observed at distal tibia.

**Table 5:** *R*^2^ Values for the Correlation Between Mechanical and Structural Parameters Computed from the Same 10 × 10 × 5 mm^3^ Trabecular-Bone Subregion of the Distal Tibia at Baseline for All Subjects

	*E*_11_	*E*_22_	*E*_33_	*G*_23_	*G*_31_	*G*_12_
BV/TV	0.71	0.44	0.92	0.69	0.81	0.66
S/C	0.76	0.45	0.85	0.68	0.82	0.62
EI	0.64	0.35	0.74	0.54	0.72	0.50
BV/TV and S/C	0.80	0.47	0.96	0.74	0.88	0.69
BV/TV and EI	0.77	0.44	0.95	0.71	0.87	0.66

All correlations were significant (*p* < .0001).

## Discussion

The results from this work show that µMRI-based structural images from trabecular and cortical bone at the distal extremities acquired in patients are suited to derive finite-element models for estimating the mechanical consequences of estrogen loss following menopause and estrogen supplementation. This study is only one of a small number of in-vivo image-based studies compellingly showing not only the structural but also mechanical manifestations of intervention. A prior study involving a smaller number of subjects had provided evidence of the architectural(18) and, subsequently, mechanical effects of testosterone replacement in men with hypogonadism.(28) Unlike in that work, the effects expected here were smaller in consideration that the women studied were early postmenopausal (mean approximately 1 year from last menstrual period), in contrast to testosterone-deficient men who had been hypogonadal for very long times and therefore eliciting a very strong treatment response. Further, this work goes considerably beyond the prior pilot study in that we performed subregional µFE analysis of a small cuboid trabecular subregion (which, however, was larger than in ref 28)—10 × 10 × 5 mm^3^ versus 5 × 5 × 5 mm^3^—but also examined the role of the cortex by performing full cross-sectional stiffness analysis similar to that described by MacNeil and Boyd.(40) Further, we studied the hypothesized mechanical treatment effects at both the distal tibia and the radius, that is, a load-bearing and a non-load-bearing anatomic site. We further brought advanced registration methods to bear for mutual alignment of the images at the three time points and with the scanner coordinate frame.(33)

In the tibia, the subregion analysis revealed significant reductions in all six moduli in the control group (ie, women who elected not to take estrogen) ranging from 3% to 5% from baseline after 24 months (*p* < .05 to <.005), whereas only three of the moduli were significantly lower after 12 months. In contrast, in the estradiol group, all six mechanical constants remained unaltered at both follow-up time points. Between-group changes in the elastic moduli, in contrast to whole-section axial stiffness values, were not significant. These results are somewhat at variance with the structural changes reported for the same cohort of patients in ref. (20). This discrepancy is likely due to the constraints imposed by the mechanical analysis in the choice of the subvolume in the present work (10 × 10 × 5 mm^3^ parallelepiped at the centroid versus larger irregularly shaped region for prior structural analysis in ref 20). It is interesting to note, however, that despite the highly significant decrements in the mechanical constants in the control subjects, BV/TV decreased only marginally at 24 months (−1.8%, *p* = .03), whereas the reduction at 12 months did not reach significance (−1.0%, *p* = .07). The data thus suggest that the predicted changes in elastic and shear moduli are largely the result of structural changes, notably the observed reduction in plate-to-rod ratio observed previously in the data.(20) Similar topologic changes had been previously observed in paired biopsies following early menopause.(41)

Of particular relevance are the effects of intervention on whole-section axial stiffness at both anatomic sites. In the tibia, women in the control group suffered a loss in estimated stiffness of about 4% within a year (*p* < .005) while gaining over 6% in response to estrogen supplementation during the same period (*p* < .05). In the radius, whole-section axial stiffness declined by about 4% during the initial 12 months (*p* < .005) and increased marginally in the estradiol group (by about 2.5%, *p* < .05). Of significance further are the large intergroup differences in change from baseline: 10% after 12 months in the tibia (*p* = .0001) and 6% in the radius (*p* < .0005).

The effect of estradiol is clearly greater at the tibia than at the radius, suggesting that the load-bearing site may be more sensitive to antiresorptive treatment. Possible synergistic effects of antiresorptive agents with mechanical stimulation have been studied widely (see, for example, ref (42)). However, most work relies on studies in animals. For example, Westerlind and colleagues(43) found that estrogen administration to ovariectomized rats reduced bone loss in the unloaded and prevented loss in the loaded limb following unilateral sciatic neurotomy. Tromp and colleagues(44) also found an interaction between estrogen and mechanical loading on bone in the ovariectomized rat model.

The highly significant between-group changes from baseline in whole-section axial stiffness at both anatomic sites emphasize the role of the cortex as being affected by estrogen depletion and supplementation. The findings are counter to the notion that short-term remodeling changes after menopause (both loss owing to reduction in estrogen levels or accrual from estrogen supplementation) affect primarily trabecular bone, which remodels faster than cortical bone. Further, the within-group increases in trabecular bone elastic and shear moduli, as well as whole-section axial stiffness in the estradiol group suggest that estrogen supplementation not only preserves but actually improves bone mechanical competence, in contrast to prior structural studies indicating mere preservation of architecture.(45) Nevertheless, the anabolic effects of estrogen replacement are well documented. Khastgir and colleagues found in older women large increases in trabecular bone volume and bone mineral density (BMD). as well as increased wall thickness, in response to estrogen supplementation.(46) Similarly, based on paired bone biopsies obtained before and after 2 years of hormone replacement in postmenopausal women, Vedi and colleagues demonstrated significantly higher cortical width.(47)

The lack of further significant gains from 12 to 24 months is somewhat puzzling but parallels the structural observations made previously.(20) Nevertheless, in the tibia, four of the elastic moduli that had not changed significantly after 12 months were all lower after 24 months (Fig. 2). In contrast, there was no further decline in whole-section stiffness after 24 months and only an insignificant further increase in the estradiol group. There are several possible reasons for these observations. First, lifestyle changes in the control group could have mitigated further declines in structural and mechanical parameters. Second, in these very early postmenopausal women, the degradation in bone quality is likely to be largest early after the onset of menopause. Third, the number of subjects proceeding to the 24-month time point was lower than those who were examined at baseline.

This study has limitations because it ignores effects of treatment on the intrinsic bone tissue properties. Paschalis and colleagues(2) found in early postmenopausal women that 2 years of estrogen/progestin supplementation increased mineral/matrix ratio, mineral crystallinity/maturity, and the relative ratio of collagen cross-links, clearly showing that antiresorptive treatment affects material properties. The same group recently has provided evidence that subjects with vertebral fractures differ in their matrix and mineral composition (ie, collagen maturity, mineral/matrix ratio, and crystallinity).(48) However, the work did not provide data on structural group differences, and BMD was matched at the proximal femur rather than at the vertebrae.

As with all CT-based µFE work using in vivo structural images to create the FE model.(49,50) our model analyzes the structural implications only of longitudinal changes on mechanical parameters. While CT can, in principle, make use of variations in mineralization density, such differences are not distinguishable from partial-volume averaging in the limited spatial resolution regime of in vivo imaging, where resolution is comparable with trabecular thickness, nor would CT be able to quantify changes in matrix chemistry. Also, with few exceptions,(36) all prior in vivo µFE work was based on thresholded images in that a fixed tissue modulus was given to the voxels assigned to bone. We found in preliminary work grayscale-based µFE analyses to be more accurate than those relying on binarized images,(51) which is what prompted us to avoid binarization, since it is well known to be fraught with error at the resolution achievable by in vivo structural imaging of trabecular bone. Finally, the study is limited to the linear regime in that ultimate strength was not evaluated, which could be obtained from the present data by invoking the Pistoia criterion,(52) which suggests that failure will occur once 2% of the bone tissue is strained beyond 0.7%.(52) Nevertheless, there is substantial evidence that yield strength and stiffness are highly correlated (*r*^2^ > 0.95; see, for example, ref. (36)).

In conclusion, MRI-based µFE analysis is able to predict the short-term mechanical implications of drug intervention of both trabecular and cortical bone. The data provide evidence of an anabolic effect of estrogen supplementation in terms of whole-section axial stiffness at both the distal tibia and radius, but the effects, in terms of both reduction in mechanical competence in the absence of estradiol administration and the observed increases in subjects receiving hormone supplementation, were greater at the tibia than at the radius.
